# Recent advances in antibacterial coatings for orthodontic appliances

**DOI:** 10.3389/fbioe.2023.1093926

**Published:** 2023-02-01

**Authors:** Nannan Wang, Jingjing Yu, Jiarong Yan, Fang Hua

**Affiliations:** ^1^ The State Key Laboratory Breeding Base of Basic Science of Stomatology (Hubei-MOST) and Key Laboratory of Oral Biomedicine Ministry of Education, School and Hospital of Stomatology, Wuhan University, Wuhan, China; ^2^ Center for Orthodontics and Pediatric Dentistry at Optics Valley Branch, School and Hospital of Stomatology, Wuhan University, Wuhan, China; ^3^ Center for Evidence-Based Stomatology, School and Hospital of Stomatology, Wuhan University, Wuhan, China; ^4^ Division of Dentistry, School of Medical Sciences, Faculty of Biology, Medicine and Health, University of Manchester, Manchester, United Kingdom

**Keywords:** orthodontics, brackets, archwires, clear aligners, antibacterial, nanoparticles, coating

## Abstract

In the process of orthodontic treatment, the presence of orthodontic appliances makes it difficult to clean tooth surfaces. This can lead to an increased level of bacterial colonization, resulting in enamel demineralization and periodontal diseases. Considering the large surface area that orthodontic appliances usually have and that they can be in direct contact with bacteria throughout the treatment, modifications in the form of coatings on the surface of orthodontic appliances can be an effective and practical approach to reducing bacterial proliferation and preventing relevant adverse effects. In this mini-review, we discuss various antibacterial coatings which have been applied onto orthodontic appliances in recent 5 years, as well as their antibacterial mechanisms and methods for the preparation of these coatings. From this mini-review, both orthodontists and researchers can get the latest findings in the field of antibacterial coatings onto orthodontic appliances, which is helpful for the decision-making in clinical practice and research activities.

## 1 Introduction

Orthodontic appliances in orthodontic treatment changes the ecological environment of the oral cavity, resulting in a significant increase in the number of cariogenic bacteria such as *Streptococcus mutans* (*S. mutans*) and *Lactobacillus* (Liu Y et al., 2017). The imbalance in oral homeostasis can lead to enamel demineralization, periodontitis and other bacteria-related adverse effects of orthodontic treatment (Hua et al., 2020). According to evidence-based clinical assessments, once enamel demineralization is formed it can hardly be fully reversed using currently available remineralizing agents (Hua et al., 2018; Hu et al., 2020). Thus, several antibacterial strategies have been used clinically to prevent enamel demineralization, including the use of antibacterial mouthwashes and toothpastes. However, these conventional interventions are largely dependent on perfect compliance from the patients, which does not exist for most of the time.

To overcome the above-mentioned problems, researchers have made efforts to add antibacterial capacity to orthodontic appliances (Zhang et al., 2018) and bonding systems (Yan et al., 2022). This can have a long-lasting effect in the patient’s mouth so as to inhibit the adhesion and growth of pathogenic bacteria without patients’ compliance. But adhesives only exist at the interface between bracket base and enamel surface, therefore the actual antibacterial effects of modified adhesives are bound to be limited. In contrast, modification of orthodontic appliances themselves, which makes use of all appliance components including brackets, archwires and clear aligners, is more promising in achieving adequate, long-term antibacterial effects (Park et al., 2018; Salehi et al., 2018; Selvaraj et al., 2021).

During recent years, increasing attention has been paid to the modification of orthodontic brackets, archwires and aligners *via* surface coatings. Materials such as metals oxides, metal element (Ghasemi et al., 2017), organic compounds (Park et al., 2018) and others (Dai et al., 2022) have been used to form such antibacterial coatings, which can lower the dependence on oral hygiene maintenance, decrease the roughness of appliance surfaces (and the resultant bacterial adhesion), and increase the biocompatibility of orthodontic appliances (Liu J M et al., 2017; Hammad et al., 2020; He et al., 2020).

Several relevant reviews have been published, but with different emphasis. Yun et al. (2022) mainly introduced the application of antibacterial nanoparticles in orthodontic materials. Arango et al. (2013) mainly summarized the coating and surface treatment methods of orthodontic metal materials. Although Bącela et al. (2020) introduced the functional coating of orthodontic archwires and its preparation method in detail, this article did not include the application of coating in other orthodontic appliances (e.g. clear aligners) and relevant research progress in the past 2 years.

Therefore, the present review aims to summarize recent research with regard to the modification of orthodontic appliances through antibacterial coatings. By briefly introducing the antibacterial mechanism of coatings, the classification of antibacterial coating materials and their application in orthodontic devices, as well as the preparation methods of coatings applied in the field of orthodontics, this mini-review is helpful for orthodontists and researchers to quickly grasp the latest findings in this area, and make accurate decisions in clinical practice and research activities.

## 2 Antibacterial mechanism of coating materials

### 2.1 Antimicrobial mechanism of nanoparticles

#### 2.1.1 Reactive oxygen species (ROS) induced oxidative stress

Nanoparticles (NPs) react with oxygen molecules to produce different types of ROS. For instance, titanium dioxide is a semiconductor that can be activated by photons with wavelengths below about 385 nm, or UVA, which are photocatalyzed to allow electrons to move freely within the conduction band (Bono et al., 2021). Oxygen and water can trap charges and produce reactive oxygen species, such as superoxide anions (O^+^_2_^−^•) and hydroxyl radicals (•OH), which degrade cell walls and plasma membranes, leading to leakage of cell contents and bacterial death. In addition, the ROS produced can diffuse into bacteria, attack proteins and inhibit the activity of certain surrounding plasmids that maintain the normal morphology and physiological processes of bacterial cells.

#### 2.1.2 Dissolved metal ion

The positively charged metal ions of NPs are released and bind to the negatively charged functional groups of the bacterial cell membrane, resulting in the chaotic dispersion of the originally ordered and tightly spaced cell membrane, resulting in the death of the bacteria. In addition, some metal ions can directly interact with functional groups of proteins and nucleic acids. For example, zinc ions have specific affinity for mercaptan groups and can be oxidized by mercaptans as inhibitors of glycolytic enzymes (Liao et al., 2020).

### 2.2 Antimicrobial mechanism of non-nanoparticles

#### 2.2.1 Reduce bacterial adhesion

Under physiological conditions, some organic compounds, such as polyethylene glycol, can provide a hydrophilic environment on the substrate surface by hydrogen bonding with water molecules, forming a stable thin water layer, interfering with the interaction between the substrate surface and the biofilm, which can effectively prevent bacterial adhesion (Peng et al., 2017).

#### 2.2.2 Contain antibacterial functional groups

Materials can be endowed with antibacterial properties by adding functional groups such as quaternary ammonium cation (QAC) (Makvandi et al., 2018) and long alkyl chain (LAC) (Park et al., 2022). Although the detailed mechanism of the antimicrobial action of QACs has not been determined, its antibacterial effect is related to the strong affinity and destructive interaction between the positively charged QACs ions and the negatively charged acidic phospholipid head groups in the microbial membrane. Long lipophilic alkyl chains penetrate bacterial cell membranes by binding to cell wall components, leading to cytoplasmic material leakage, bacterial autolysis, and cell death.

## 3 Antibacterial coating materials

### 3.1 Antibacterial coating materials for fixed appliances

#### 3.1.1 Metals and their compounds

A number of different metals and their compound materials have been coated onto fixed orthodontic appliances, including silver (Ag), titanium dioxide (TiO_2_), zinc oxide (ZnO), zirconium oxide (ZrO_2_), and titanium nitride (TN). Among these, TiO_2_ is most widely studied and utilized.

Silver nanoparticles (Ag NPs), have become common materials in the fields of dentistry and orthodontics due to their high antibacterial activity. Studies have shown that without changing the physical and chemical properties of archwires, the silver nanoparticle coated Abzil^®^ archwires showed a significant reduction in the presence of *S. aureus* and S. mutans, while Orthometric^®^ archwires did not (Goncalves et al., 2020). The nano-silver coated bracket shows great antibacterial characteristic against *S. mutans*, and its contact inhibition feature is effective especially in the reduction of smooth surface caries around the brackets (Ghasemi et al., 2017; Metin-Gürsoy et al., 2017).

The nano-silver coatings for brackets can be obtained by physical vapor deposition (PVD), galvanic silver and plasma immersion ion implantation and deposition (PIIID). For the antibacterial effect, there’s no significant difference between the PIIID procedure with silver ions and a PVD or galvanic silver coating (Meyer-Kobbe et al., 2019).

The combination of TiO_2_ and Ag NPs can compensate for the weakness in the decreased antibacterial property due to insufficient light in the mouth. A combination of Ag and TiO_2_ for the coating onto SS brackets showed excellent anti-adherent properties for the major oral pathogens *S. mutans* and Porphyromonas gingivalis (*P. gingivalis*). Ag NPs can also be combined with ZnO NPs. The Ag/ZnO coated brackets exhibit the highest antibacterial effect in comparison with Ag and ZnO individually coated brackets on *S. mutans* and *L. acidophilus*, and the antibacterial activity of the bracket coatings can persist over time (Zeidan et al., 2022).

TiO_2_ has attracted much attention because of its excellent antibacterial activity and biocompatibility (Mollabashi et al., 2020; Venkatesan et al., 2020). There are two phases of TiO_2_, the anatase phase and the rutile phase, and they both exhibit significant antibacterial characteristics. The unwanted cytotoxicity is also seen in these phases—moderate to severe in the rutile phase, but only mild in the anatase phase (Baby et al., 2017). Thus, it is recommended that anatase phase TiO_2_ be coated onto brackets, so that antibacterial property is obtained while being only slightly cytotoxic at the same time. A clinical trial showed that TiO_2_ coated archwires can effectively reduce the adherence of *S. mutans* during the initial stage of orthodontic treatment (Mollabashi et al., 2020).

The antibacterial effect of TiO_2_ is achieved through the process of photocatalysis in the ultra violet (UV) region (<380 nm) (Salehi et al., 2018). The way of doping and surface modification allows TiO_2_ to exhibit catalytic activity within the visible light region, consequently improving the photocatalytic efficiency of TiO_2_ (Salehi et al., 2018). For instance, Nitrogen-doped TiO_2_ (N-doped TiO_2_) can expand the absorption edge to the visible region and narrow the band gap (Amini et al., 2017), showing the characteristic of exceptional visible light and UV light activities (Asahi et al., 2001). Under the condition of ultraviolet filtration, the bactericidal rate of N-doped TiO_2_ coated composite archwires (CAWs) and TiO_2_ coated CAWs were 87.2% and 5.9% respectively (Liu J M et al., 2017). Brackets coated with N-doped TiO_2_ show a significant antibacterial effect against *S. mutans*, *L. acidophilus*, *A. viscous*, and *C. albicans* (Salehi et al., 2018). Also, this longer lasting antibacterial effect for TiO_2_ coated brackets and may not decrease over time, regardless of whether the illumination time was 24 h or 60 m (Salehi et al., 2018).

Another metallic oxide material coated onto orthodontic appliances is ZnO. ZnO nanoparticles (ZnO NPs) have photocatalytic bactericidal activity, wide antibacterial spectrum and low drug resistance (Gharpure and Ankamwar 2020; Pushpalatha et al., 2022). The effect of coating five kinds of ZnO NPs onto NiTi archwires on inhibiting S. mutans was studied (Gholami et al., 2021). The results indicated that chemical vapor deposition (CVD) method had the highest bacteriostatic rate of 98%. ZnO coated brackets could exhibit enhanced antibacterial effect against *S. mutans* and *L. acidophilus* in comparison to the uncoated (Zeidan et al., 2022). The carbon quantum dots (CQDs) is a kind of unique materials with the upconversion fluorescence property, which can convert visible light into UV or near-UV light. Combining the antibacterial ability of ZnO and the upconversion fluorescence property of CQD, the ZnO/CQD composite coating exhibits great antibacterial activity under natural visible light (Zhang et al., 2018).

ZrO_2_ nanoparticles (ZrO_2_ NPs) have excellent biocompatibility and good adhesion on metal surfaces. ZrO_2_ NPs coated archwires showed good antibacterial activity against *S. mutans* and *S. aureus*. The antibacterial activity increased with the increase of corresponding concentration, but the inhibition on *C. albicans* was the same at different concentrations (Selvaraj et al., 2021).

Besides metallic oxide, TN is another metallic compound that has been used. The TN coating has been in use for implants since 2000 (Jabbari et al., 2012) while in orthodontic field, TN coatings onto the surface of SS brackets have no influence on the formation of the *S. mutans* biofilm and do not reduce the growth of *S. mutans*.

#### 3.1.2 Organic materials

The SS archwires coated with polyethylene glycol (molecular weight 5,000) have good anti-bacterial adhesion performance, which can inhibit the growth of bacteria up to 10 h. (Peng et al., 2017). Lysozyme selectively breaks down the cell wall of microorganisms without destroying other tissues, and was used for antibacterial modification of archwires. The 40 g/L lysozyme coating on the CAWs had the strongest inhibitory effect on *S. aureus* (He et al., 2020).

#### 3.1.3 Other materials

Graphene oxide (GO) is an oxide of graphene, which has increased oxygen-containing functional groups, making its properties more active than graphene. Dai et al. (2022) found that with the increase of graphite oxide concentration, the bacteriostatic effect of GO coating was enhanced. *In vitro* and *in vivo* studies have been conducted to compare the antibacterial characteristics for different coatings onto orthodontic appliances (Table 1).

**TABLE 1:** Comparison of the antibacterial characteristics for different coatings onto orthodontic appliances.

No.	Authors	Study type	Coating materials	Orthodontic appliances and their material(s)	Coating method(s)	Antibacterial activity	Effectiveness
1	Mollabashi et al. (2020)	clinical study	Metallic compound (TiO2 NPs)	SS wires	PVD	S. mutans	CFU/mL: coated: 297.7 ± 581.7, uncoated: 1,141.8 ± 2,108.4 (*p* = 0.005)
2	Venkatesan et al. (2020)	clinical study	Metallic compound (TiO2 NPs)	NiTi wires	PVD (the RF magnetron sputtering method)	S. mutans	Ct values: control: 30.97 ± 2.23; NiTi: 37.00 ± 1.90 (*p* = 0.0005)
3	Amini et al. (2017)	*in vivo*	Metallic compound (TiO2)	SS wires	PVD	S. mutans	CFU: control: 8 ± 7.4 × 104; experimental group: 4 ± 3.4 ×104
4	Salehi et al. (2018)	*In vitro*	Metallic compound (N-doped TiO2)	SS brackets	The RF magnetron sputtering method	S. mutans	CFU/µm3: nano-TiO2, 37.82 ± 5.15; control, 401.21 ± 13.72 (*p* < 0.001)
5	Liu J M et al. (2017)	*In vitro*	Metallic compound (N-doped TiO2)	new CAWs	PVD (the RF magnetron sputtering method)	S. mutans	Reduction rate: 87.2%
6	Gholami et al. (2021)	*In vitro*	Metallic compound (ZnO NPs)	NiTi wires	CVD, CP, polymer composite, the S-G dip coating method, electrospinning	S. mutans	Reduction (%): CVD: 98.61; S-G: 93.05; electrospinning: 72.07; chemical: 96.14; polymer: 89.97
7	Hammad et al. (2020)	*In vitro*	Metallic compound (ZnO NPs)	NiTi wires	Electro-chemical precipitation	*S. aureus*, S. pyogens, *E. coli*	Mean diameter of inhibition zones: *S. aureus*: 4.25; S. pyogens: 6.25; *E. coli*: 3.57 (*p* < 0.001)
8	Zhang et al. (2018)	*In vitro*	Metallic compound (ZnO/CQDs composite coating)	SS brackets	The high vacuum magnetron sputtering coating method	*E. coli*, S. mutans, *S. aureus*	The antibacterial rate: ZnO, 57.14%, 45.31%, 42.4% respectively; ZnO/CQDs, 92.35%, 96.13%, 90.28% respectively (*p* < 0.01)
9	Selvaraj et al. (2021)	*In vitro*	Metallic compound (ZrO2 NPs)	SS and NiTi wires	Thermal evaporation	*Lactobacillus*, S. mutans, S. aureu, C. albicans	Mean diameter of inhibition zones/mm: S. mutans: 11 to 15; S. mutans: 9 to 12; C. albicans: 9; *Lactobacillus*:/
10	Teixeira et al. (2021)	*In vitro*	Metallic compound (TN and TNCP)	SS brackets	The cathodic cage deposition	S. mutans	No significant differences as to the reduction on the growth of S. mutans
11	Goncalves et al. (2020)	*In vitro*	Metal element (Ag NPs)	SS wires	Hydrothermal synthesis	*S. aureus*; S. mutans	Bacterial density: Orthometric^®^. *S. aureus*: 174 ± 0.04; S. mutans: 0.118 ± 0.007; Abzil^®^. *S. aureus*: 0.113 ± 0.01; S. mutans: 0.118 ± 0.009 (*p* < 0.05)
12	Meyer-Kobbe et al. (2019)	*in situ*	Metal element (Ag-coated and Ag ion-implanted)	SS brackets	The PIIID procedure, PVD coating and galvanic coating	initial intraoral biofilm	Biofilm volume/µm3: control, 7.24 × 108 ± 3.11 × 108; galvanic silver, 2.62 × 107 ± 4.81 × 107; PVD, 4.44 × 107 ± 9.06 × 107; PIIID,3.82 × 107 ± 7.53 × 107 (*p* ≤ 0.05)
13	Zeidan et al. (2022)	*in vitro*	Metal element (Ag/ZnO nanocomposite)	SS brackets	PVD	S. mutans, L. Acidophilus	Rate of survival of the S. mutans bacterial cells: control, 1403.75 ± 4.20% CFU; Ag, 1016.25 ± 2.80% CFU; ZnO, 1157.50 ± 5.40% CFU; Ag/ZnO, 767.50 ± 9.60% CFU (*p* < 0.05)
14	Zhang et al. (2020)	*in vitro*	Metal element (AuDAPT)	Invisalign aligner	Oxygen plasma treatment, and then soaking	P. gingivalis	The SEM images confirmed that fewer P. gingivalis were live on the AuDAPT-coated Invisalig, and biofilm did not form compared to that on untreated substrates
15	Xie et al. (2020)	*in vitro*	Metal element (QA-GNCs)	Invisalign aligner	Oxygen plasma treatment, and then soaking	S. mutans	The time-kill kinetics of QA-GNCs (5 μg/mL) was comparable to that of vancomycin (5 μg/mL) during the exponential growth phase
16	He et al. (2020)	*In vitro*	Organic materials (lysozyme)	New CAWs	LPD	*S. aureus*	Live/dead bacteria ratios: 59%–82%
17	Peng et al. (2017)	*In vitro*	Organic materials (polyethylene glycol-grafted dental material)	SS wires	silanization	S. mutans	Bacteral density: control: 9.03 × 105 cm−2; experimental group: 4.64 × 105 cm−2
18	Park et al. (2018)	*In vitro*	Organic materials [(CMC/CHI)20 nanofilms]	PETG COA	LbL assembly and cross-linking	biofilm adhesion	The ATP luminescent signal of the coated PETG is ∼4 times lower than that of the bare PETG, which indicates that the coated PETG exhibits low bacterial viability
19	Park et al. (2022)	*in vitro and in vivo*	Organic materials (PSQ)	COA	Oxygen plasma treatment, and then soaking	*S. aureus* and *P. aeruginosa*	The formation and maturation of dental plaque was inhibited in the nanoscale PSQ-coated COAs compared to the bare COAs
20	Dai et al. (2022)	*In vitro*	Other materials (GO)	NiTi wires	Silane coupling	S. mutans	Reduction rate: 23%–77%

TiO2, titanium nitride; TN, titanium nitride; TNCP, titanium nitride doped with calcium phosphate; Ag, silver; AuDAPT, 4,6-diamino-2-pyrimidinethiol-modified gold nanoparticles; QA-GNCs, quaternary ammonium-modified gold nanoclusters; PSQ, robust polysilsesquioxane; GO, graphene oxide; PVD, physical vapor deposition; PIIID, plasma immersion ion implantation and deposition; CVD, chemical vapor deposition; RF, radio frequency; VTE, vacuum thermal evaporation; S-G, sol gel; LPD, liquid phase deposition; ZrO2, zirconium oxide; NPs, nanoparticles; ZnO, zinc oxide; CQDs, carbon quantum dots; N, nitrogen; NiTi, nickel titanium; SS, stainless steel; CAWs, composite arch wires; COAs, clear overlay appliances; CMC, carboxymethyl cellulose; CHI, chitosan; LBL, layer-by-layer; PETG, polyethylene terephthalate glycol.

### 3.2 Antibacterial coating materials for clear aligners

Organic substances, such as carboxymethyl chitosan, and inorganic substances, such as gold nanoparticles were used as the coating materials of clear aligners (Park et al., 2018; Xie et al., 2020). Park et al. (2018) used carboxymethylcellulose (CMC) and chitosan (CHI) to fabricate polysaccharide-based antibacterial coating on polyethylene terephthalate glycolmodified (PETG) which is a normal clear aligner raw material. The coating formed a superhydrophilic surface in aligners and could significantly reduce the adhesion of bacteria. Polysilsesquioxane (PSQ) was also used for the modification of clear aligners. Clear aligners were coated with a ladder-like PSQ containing quaternary ammonium cations and long alkyl chains, and the antibacterial ability of the PSQ-coated aligners were investigated *in vitro* and *in vivo*. PSQ-coated aligners could inhibit the growth of *S. aureus* and *Pseudomonas aeruginosa* (*P. aeruginosa*), and could reduce the dental plaque formation on beagle dogs (Park et al., 2022).

Xie et al. (2020) modified clear aligners with quaternary ammonium -modified gold nanoclusters (QA-GNCs). The QA-GNCs coated aligners could prevent the adhesion of *S. mutans* and biofilm formation on the aligner, and this antibacterial effect could last for 3 months. Another gold nanocomposites, 4,6-diamino-2-pyrimidinethiol-modified gold nanoparticles (AuDAPT), was also used for the coating modification of clear aligners. The coated aligners showed significant antibacterial ability against *P. gingivalis* and maintained the biocompatibility (Zhang et al., 2020).

## 4 Methods for preparing antibacterial coatings

The preparation methods of coating mainly include physical deposition processes and chemical deposition processes. Physical deposition processes include evaporation and sputtering (magnetron, radio frequency, and high-energy ion scattering). Chemical deposition processes can be divided into vapor deposition (electrochemical deposition and atomic layer deposition) and liquid deposition (sol-gel method, dip coating method, spin coating and spray pyrolysis). The following are some coating preparation techniques that have been used in orthodontics.

### 4.1 Physical vapor deposition method

Physical vapor deposition (PVD), refers to the use of thermal evaporation or glow discharge, arc discharge and other physical processes to change the phase of the material under vacuum conditions. The surface of SS and NiTi archwires could be modified by PVD of silver. As a subcategory of PVD, the radiofrequency (RF) magnetron sputtering method is the most commonly used one for coating TiO_2_ onto SS brackets (Baby et al., 2017). The magnetron sputtering method can also be used to coat the ZnO layers in high vacuum, after the prepared CQDs solution drops on SS brackets, consequently forming the ZnO/CQDs composite coating (Zhang et al., 2018).

### 4.2 Chemical vapor deposition method

Chemical vapor deposition (CVD) is a process in which chemical substances in vapor state are reacted and deposited on the surface of substrate by heating or plasma excitation to form the required solid coating. The size of ZnO NPs formed by CVD is uniform in the range of 59–61 nm, and the coating on the NiTi archwires have high density and good dispersion (Gholami et al., 2021). Nearly all studies on the modification of clear aligners used coatings synthesized by CVD (Park et al., 2018; Xie et al., 2020; Zhang et al., 2020; Park et al., 2022).

### 4.3 Chemical precipitation method

The chemical precipitation method points to the method of adding chemical agent to the target metal solution to be loaded, controlling a certain temperature and pH value, so that the object metal is deposited on the surface of the carrier. ZnO NPs formed by dropping ammonia water into a zinc nitrate solution to make it alkaline and under intense mixing with or without the action of an external electric field (Hammad et al., 2020). The results show that the ZnO coating is hexagonal with spherical ends and uniform dispersion, and the diameter is in the range of 30–150 nm (Gholami et al., 2021). The antibacterial rate of NiTi archwires obtained by this method can reach 96.14%.

### 4.4 Sol-gel method

By sol-gel film dipping method NiTi archwires could be coated with ZnO NPs (Gholami et al., 2021). Compared with other methods, Ag/ZnO NPs formed by sol-gel method have smaller sizes, larger surface volume ratio and higher antibacterial activity.

## 5 Discussion

The presence of orthodontic appliances leads to an imbalance in the oral environment and an increase in the number of pathogenic bacteria associated with enamel demineralization and periodontitis (Liu Y et al., 2017). However, antibacterial mouthwash or toothpaste cannot provide long-term sustained antibacterial effect, and modified adhesives existing between the base of the bracket and the surface of the enamel only have limited antibacterial effect. Therefore, in order to provide long-term sustained antibacterial effect, antibacterial modification of orthodontic appliances themselves is warranted.

**FIGURE 1:**
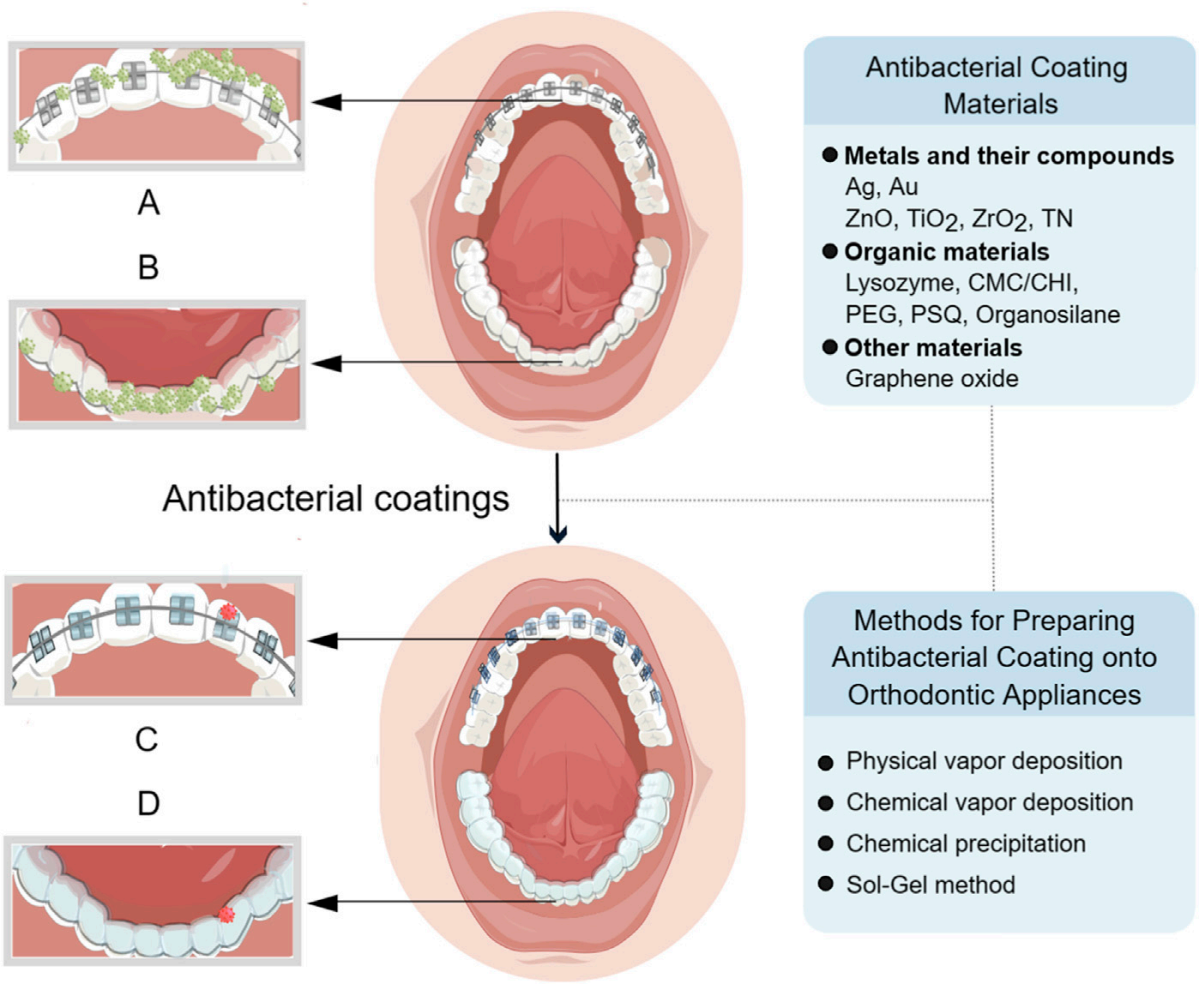
**(A)** Bacteria build up on the surface of fixed appliance before the application of coating. **(B)** Bacteria build up on the surface of clear aligner before the application of coating. **(C)** Bacteria on the fixed appliance are reduced after coating. **(D)** Bacteria on the clear aligner are reduced after coating. Created with FigDraw.com.

Based on recently published research findings, this review introduced the latest progress in antibacterial coatings for orthodontic appliances, including the antibacterial mechanism of coatings, the classification of antibacterial coating materials onto orthodontic appliances, as well as the preparation method of coatings applied in the field of orthodontics (Figure 1), which is helpful for orthodontic clinicians to fully understand the classification of each type of coating materials and their application in orthodontic appliances.

However, there is still much to be studied about the anti-bacterial coatings onto orthodontic appliances. Firstly, the antibacterial, mechanical or physical properties were only studied in a relatively short period of time, while orthodontic materials still need to be evaluated for long-term effects. Secondly, the majority of the studies included in this article are *in vitro* experiments, while their effects may not be practical for *in vivo* applications, as the oral cavity is a complex environment with continuous changes in pH, saliva flow, and food chemicals. Thirdly, in addition to antibacterial properties, other properties of orthodontic appliance coatings such as friction reduction, advanced mechanical properties and biocompatibility are also of concern for such research. Further studies are needed to investigate the correlation between anti-bacterial properties and other properties of orthodontic appliances. Coating materials can also cause the problem of allergy to metallic elements such as nickel ions and even the toxic effects of silver ion. Therefore, more attention should be paid to the release of nickel ions and the safety of silver irons.

Clear aligner is the new trend of orthodontic appliances. However, there are few studies on the application of NPs in clear aligners for microbial inhibition at present, and more attention should be paid to antibacterial NPs combined with clear aligners. In addition, although a number of materials and techniques has been implemented to modify the surfaces of dental materials, only a few are actually used in orthodontic clinics, especially in areas such as friction control and reduction of bacterial adhesion. Future studies are needed to validate the effects of existing *in vitro* findings in clinical practice.
